# Association between physical activity and psychological resilience in older adults: an analysis of a prospective cohort study in China

**DOI:** 10.3389/fpsyg.2026.1958017

**Published:** 2026-10-09

**Authors:** Haozheng Xu, Jiahao Wang, Yuxiang Yang, Haiwei Li

**Affiliations:** School of Physical Education, Shanxi Normal University, Taiyuan, China

**Keywords:** Chinese elderly, CLHLS, exercise, physical activity, psychological resilience

## Abstract

**Background:**

Although numerous studies have confirmed the mental health benefits of physical activity, evidence on the association between physical activity and psychological resilience—particularly longitudinal evidence that accounts for dynamic patterns of activity change—remains limited. Therefore, this study aimed to examine the cross-sectional and longitudinal associations between physical activity patterns and psychological resilience among older adults in China.

**Methods:**

This study used two waves of data (2011–2014) from the Chinese Longitudinal Healthy Longevity Survey (CLHLS) and included Chinese older adults, and employed both cross-sectional and prospective analysis designs. Physical activity was categorized into four patterns of change: consistently inactive, active-to-inactive, inactive-to-active, and consistently active. Psychological resilience was assessed using a five-item scale and dichotomized as high or low. Logistic regression and Cox proportional hazards models were used to examine the associations between physical activity patterns and psychological resilience, adjusting for sociodemographic characteristics, lifestyle factors, and health status.

**Results:**

In the cross-sectional analysis, compared with the remain-inactive group, the inactive-to-active group (OR = 0.59, 95% CI: 0.51–0.69, *p* < 0.001) and the remain-active group (OR = 0.61, 95% CI: 0.51–0.72, *p* < 0.001) had lower odds of low PR, whereas the active-to-inactive group was not significantly associated with low PR (OR = 0.94, 95% CI: 0.77–1.13, *p* = 0.486). In the longitudinal analysis, the active-to-inactive (HR = 0.73, 95% CI: 0.56–0.96, *p* = 0.023), inactive-to-active (HR = 0.72, 95% CI: 0.61–0.85, *p* < 0.001), and remain-active groups (HR = 0.59, 95% CI: 0.48–0.73, *p* < 0.001) had lower hazards of incident low PR. No statistically significant PA-by-subgroup interactions were detected.

**Conclusion:**

PA patterns characterized by current activity, regardless of past activity status, were associated with lower odds of prevalent low PR and lower hazards of incident low PR among older adults. These observational findings support further investigation of PA as a potentially modifiable factor associated with psychological resilience.

## 1 Introduction

Psychological resilience (PR) generally refers to an individual’s comprehensive capacity to cope with, adapt to, and maintain a positive mindset in the face of stress or adversity (Nishi et al., 2010; Shi et al., 2019). It encompasses both a positive personal trait and a dynamic process through which individuals exhibit adaptive responses despite experiencing significant stress or trauma (Luthar et al., 2000). Higher levels of PR are associated with enhanced well-being, fewer depressive symptoms (Wermelinger Ávila et al., 2017), and greater life satisfaction (Smith and Hollinger-Smith, 2015). Evidence further indicates that older adults with higher PR report greater self-perceived successful aging (Jeste et al., 2013). Moreover, the benefits of PR extend to multiple objective health outcomes: individuals with higher PR tend to maintain better functional independence in daily life (Wang et al., 2020), exhibit superior overall health status (Rolandi et al., 2024), and demonstrate longer life expectancy and notable survival advantages (Zhang et al., 2024). The global population of older adults is projected to reach 1.4 billion by 2030 and 2.1 billion by 2050 (Smith and Wesselbaum, 2024). This demographic shift has become a major public health challenge. Consequently, understanding the factors that influence PR in older adults has substantial practical and policy relevance.

At the individual level, existing literature has identified several key factors associated with PR, including psychological factors (Bhatnagar, 2021), biological factors (Smeeth et al., 2021; Bjekić and Petrović, 2024), health behaviors (Neumann et al., 2022), and social factors (Górska et al., 2022). Among these, physical activity (PA) is defined as any bodily movement produced by skeletal muscles that results in energy expenditure (Caspersen et al., 1985), has received increasing attention as a crucial health behavior for promoting mental health in older adults. Evidence indicates that maintaining regular PA is not only associated with fewer depressive symptoms (Zhang et al., 2025) and greater life satisfaction (Oliveira et al., 2025) but is also prospectively associated with more favorable trajectories of psychological health (Steinmo et al., 2014). Furthermore, multiple intervention studies have demonstrated that structured exercise programs designed to increase PA levels effectively enhance cognitive function and overall health among older adults (Liao et al., 2025). Collectively, these findings suggest that PA may represent a feasible and important approach to improving mental health in older adults by strengthening PR.

Although numerous studies have established positive associations between PA and various mental health outcomes (Wu et al., 2025), research focusing specifically on PR remains limited. In many studies, resilience is reported as only one of several mental health outcomes, and most existing evidence relies on cross-sectional designs (O’Doherty et al., 2024), leaving the longitudinal associations and potential causal pathways between PA and PR unclear. Therefore, this study aims to systematically examine the longitudinal relationship between patterns of PA change and PR in older adults using a secondary analysis of a prospective cohort design, thereby addressing current evidence gaps and providing empirical guidance for promoting healthy aging.

## 2 Methods

### 2.1 Study design and participants

This study was a secondary analysis of data from the Chinese Longitudinal Healthy Longevity Survey (CLHLS), incorporating both cross-sectional and prospective analyses. Data were obtained from the Chinese Longitudinal Healthy Longevity Survey (CLHLS), a prospective cohort study of adults aged 65 years and older (Zeng, 2012). The CLHLS employed a multistage, stratified cluster sampling strategy and primarily recruited adults aged 65 years and older from randomly selected counties and cities in China. Eligible older adults were interviewed regardless of their health status, while individuals who did not meet the age and sampling criteria were not included in the core older-adult survey (Zeng, 2012). Initiated in 1998, the CLHLS conducts follow-up surveys every 2–3 years. Detailed information on the study design and population has been reported elsewhere. The study was approved by the Peking University Research Ethics Committee (IRB00001052-13074), and all participants or their proxies provided written informed consent. The present study was a secondary analysis of de-identified CLHLS data and did not involve additional participant contact or collection of identifiable information; therefore, additional ethical approval was not required for the current analysis.

Data were drawn from 9,765 baseline records in the 2011–2012 wave, with follow-up information from the 2014 wave. Purposive sampling was used in this secondary analysis to select eligible participants from the CLHLS database according to the prespecified inclusion and exclusion criteria. All 9,765 baseline records from the 2011–2012 CLHLS wave were assessed against the prespecified inclusion and exclusion criteria to derive the cross-sectional and longitudinal analytic samples. The cross-sectional analysis included participants aged 65 years and older with valid five-item PR scores, PA data, and complete data on all adjustment covariates. These criteria yielded a cross-sectional sample of 6,255 participants. To examine newly observed low PR at follow-up, we excluded participants with low PR at baseline (score <19, the baseline median). Of the remaining 3,850 participants, 1,341 lacked a valid 2014 PR score, leaving 2,509 for the longitudinal complete-case analysis. The participant selection process and reasons for exclusion are shown in Figure 1.

**Figure 1:**
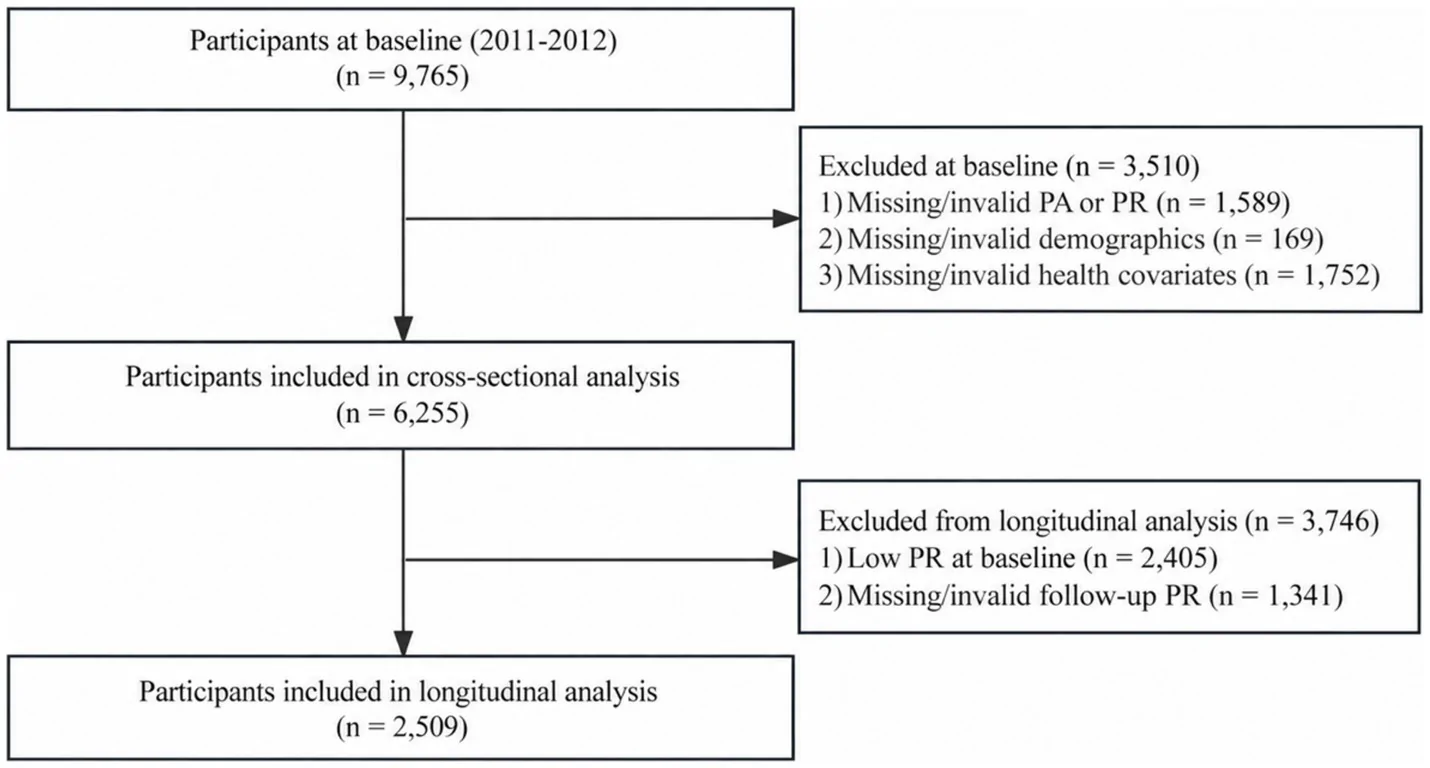
Flowchart of participant selection.

### 2.2 Assessment of PA

In the survey, participants were asked two questions: “Do you currently exercise regularly?” and “Did you exercise regularly in the past?” Both questions required binary responses of “yes” or “no.” Based on their answers, participants were categorized into four groups: (1) consistently inactive, for those who answered “no” to both questions; (2) active-to-inactive, for those who answered “no” to the first question and “yes” to the second; (3) inactive-to-active, for those who answered “yes” to the first question and “no” to the second; and (4) consistently active, for those who answered “yes” to both questions (Yin et al., 2023).

### 2.3 Assessment of PR

PR was assessed using a five-item scale. The items included: “Feel more useless as you get older,” “Look on the bright side of things,” “Feel fearful or anxious,” “Feel lonely and isolated,” and “Make your own decisions regarding personal matters.” Participants rated each item on a 5-point Likert scale (Always, Often, Sometimes, Rarely, Never), yielding a total score from 5 to 25, with higher scores indicating greater PR. PR scores were dichotomized at the median into low (below the median) and high (above the median) levels (Lou et al., 2023).

### 2.4 Covariates

In the analyses, we controlled for potential confounders associated with both PA and PR, including baseline sociodemographic characteristics, lifestyle factors, and health status. Sociodemographic variables comprised age, sex (male/female), educational attainment (illiterate/literate), residence (rural/urban), marital status (married/unmarried), living with family members (yes/no), and health insurance coverage (yes/no). Lifestyle factors included smoking status (non-smoker/smoker) and alcohol consumption (non-drinker/drinker). Health status variables, collected at baseline via self-reported questionnaires, included body mass index (BMI), diabetes (yes/no), cardiovascular disease (yes/no), cerebrovascular disease (yes/no), self-rated quality of life (good/fair/poor), depressive symptoms (yes/no), and sleep quality (good/fair/poor).

### 2.5 Statistical analysis

Continuous variables were presented as mean ± standard deviation (SD) for normally distributed data, or as median with interquartile range for non-normally distributed data. Categorical variables were reported as counts and percentages. Differences in continuous variables were evaluated using analysis of variance (ANOVA), while differences in categorical variables were assessed with the chi-square test.

To examine the association between PA and PR, we employed two regression approaches. For the cross-sectional analysis, logistic regression was used to estimate odds ratios (ORs) and 95% confidence intervals (CIs) for the relationship between PA and PR. For the longitudinal analysis, Cox proportional hazards regression was applied to assess hazard ratios (HRs) and 95% CIs for the association between baseline PA and incident low PR during follow-up. Three models were fitted: Model 1 was unadjusted; Model 2 adjusted for age, sex, educational attainment, residence, marital status, cohabitation with family members, and health insurance coverage; and Model 3 additionally adjusted for smoking status, alcohol consumption, BMI, diabetes, cardiovascular disease, cerebrovascular disease, depressive symptoms, self-rated quality of life, and sleep quality.

In multivariable Cox regression models, subgroup analyses were conducted stratified by age (<90 vs. ≥90 years) (Yin et al., 2023), sex, residence, marital status, educational attainment, smoking status, alcohol consumption, BMI (<24 vs. ≥24 kg/m^2^), and the presence of chronic diseases to evaluate the association between PA and risk of low PR. Sensitivity analyses were performed by including participants with missing covariate data using multiple imputation and excluding participants with depressive symptoms at baseline to assess the robustness of the findings. All analyses were conducted using Stata 18.0 and R 4.3.3, with two-sided *p* < 0.05 considered statistically significant.

## 3 Results

### 3.1 Baseline characteristics of the study population

The cross-sectional analysis included 6,255 participants, of whom 2,405 (38.4%) had low PR. Group sizes were 3,125 for remain inactive, 677 for active to inactive, 1,367 for inactive to active, and 1,086 for remain active. Participants differed across PA groups in age, sex, residence, education, marital status, insurance, living arrangement, alcohol drinking, BMI, chronic diseases, self-rated health, depressive symptoms, sleep quality, and low PR prevalence. Current smoking did not differ significantly across groups. Detailed results are presented in Table 1.

**Table 1:** Baseline characteristics of the study population stratified by PA (cross-sectional analysis).

Variables	Total (*n* = 6,255)	Remain inactive	Active to inactive	Inactive to active	Remain active	*p*
		(*n* = 3,125)	(*n* = 677)	(*n* = 1,367)	(*n* = 1,086)	
Age (years)^a^	83.4 ± 10.9	84.0 ± 11.6	86.4 ± 10.6	82.0 ± 9.8	81.5 ± 10.0	<0.001
Gender (%)						<0.001
Males	3,092 (49.4)	1,440 (46.1)	341 (50.4)	667 (48.8)	644 (59.3)	
Females	3,163 (50.6)	1,685 (53.9)	336 (49.6)	700 (51.2)	442 (40.7)	
Residence (%)						<0.001
Rural	3,229 (51.6)	2019 (64.6)	258 (38.1)	602 (44.0)	350 (32.2)	
Town	3,026 (48.4)	1,106 (35.4)	419 (61.9)	765 (56.0)	736 (67.8)	
Education (%)						<0.001
Illiterate	3,242 (51.8)	1890 (60.5)	321 (47.4)	694 (50.8)	337 (31.0)	
Literacy	3,013 (48.2)	1,235 (39.5)	356 (52.6)	673 (49.2)	749 (69.0)	
Marital (%)						<0.001
Married	2,794 (44.7)	1,333 (42.7)	268 (39.6)	623 (45.6)	570 (52.5)	
Other	3,461 (55.3)	1792 (57.3)	409 (60.4)	744 (54.4)	516 (47.5)	
Health insurance (%)						<0.001
Yes	5,445 (87.1)	2,772 (88.7)	543 (80.2)	1,224 (89.5)	906 (83.4)	
No	810 (12.9)	353 (11.3)	134 (19.8)	143 (10.5)	180 (16.6)	
Living with family (%)						<0.001
Cohabitation	5,055 (80.8)	2,472 (79.1)	579 (85.5)	1,113 (81.4)	891 (82.0)	
Living alone	1,200 (19.2)	653 (20.9)	98 (14.5)	254 (18.6)	195 (18.0)	
Smoke (%)						0.095
Yes	1,276 (20.4)	620 (19.8)	158 (23.3)	263 (19.2)	235 (21.6)	
No	4,979 (79.6)	2,505 (80.2)	519 (76.7)	1,104 (80.8)	851 (78.4)	
Drink (%)						0.048
Yes	1,192 (19.1%)	571 (18.3%)	121 (17.9%)	296 (21.7%)	204 (18.8%)	
No	5,063 (80.9%)	2,554 (81.7%)	556 (82.1%)	1,071 (78.3%)	882 (81.2%)	
BMI (mean ± SD)	21.6 ± 4.2	21.2 ± 3.9	21.5 ± 5.0	21.9 ± 4.2	22.2 ± 4.3	<0.001
Diabetes (%)						<0.001
Yes	284 (4.5)	93 (3.0)	41 (6.1)	73 (5.3)	77 (7.1)	
No	5,971 (95.5)	3,032 (97.0)	636 (93.9)	1,294 (94.7)	1,009 (92.9)	
Heart disease (%)						<0.001
Yes	799 (12.8)	329 (10.5)	115 (17.0)	179 (13.1)	176 (16.2)	
No	5,456 (87.2)	2,796 (89.5)	562 (83.0)	1,188 (86.9)	910 (83.8)	
Cerebrovascular (%)						<0.001
Yes	483 (7.7)	215 (6.9)	69 (10.2)	90 (6.6)	109 (10.0)	
No	5,772 (92.3)	2,910 (93.1)	608 (89.8)	1,277 (93.4)	977 (90.0)	
Self-reported healthy (%)						<0.001
Good	3,063 (49.0)	1,409 (45.1)	296 (43.7)	710 (51.9)	648 (59.7)	
Average	2,279 (36.4)	1,237 (39.6)	239 (35.3)	476 (34.8)	327 (30.1)	
Poor	913 (14.6)	479 (15.3)	142 (21.0)	181 (13.2)	111 (10.2)	
Depressive (%)						0.002
Yes	984 (15.7)	483 (15.5)	140 (20.7)	207 (15.1)	154 (14.2)	
No	5,271 (84.3)	2,642 (84.5)	537 (79.3)	1,160 (84.9)	932 (85.8)	
Sleep (%)						<0.001
Good	4,019 (64.3)	1955 (62.6)	403 (59.5)	900 (65.8)	761 (70.1)	
Average	1,494 (23.9)	816 (26.1)	173 (25.6)	288 (21.1)	217 (20.0)	
Poor	742 (11.9)	354 (11.3)	101 (14.9)	179 (13.1)	108 (9.9)	
Low psychological resilience	At/Above median: 3850 (61.6) Below median: 2405 (38.4)	At/Above median: 1710 (54.7) Below median: 1415 (45.3)	At/Above median: 377 (55.7) Below median: 300 (44.3)	At/Above median: 958 (70.1) Below median: 409 (29.9)	At/Above median: 805 (74.1) Below median: 281 (25.9)	<0.001

a Continuous variables are expressed as means ± standard deviations and categorical variables are expressed as percentages. BMI, Body Mass Index.

The longitudinal analysis included 2,509 participants with baseline PR at or above the median and valid 2014 PR data. The mean age was 79.3 years, and 1,390 participants (55.4%) were male. During follow-up, 843 participants developed low PR (33.6%). Group sizes were 1,075 for remain inactive, 206 for active to inactive, 675 for inactive to active, and 553 for remain active. The incidence of low PR was 42.2%, 30.6%, 29.9%, and 22.4%, respectively. Detailed baseline characteristics are shown in Table 2.

**Table 2:** Baseline characteristics of the study population stratified by PA (longitudinal analysis).

Variables	Total (*n* = 2,509)	Remain inactive	Active to inactive	Inactive to active	Remain active	*p*
		(*n* = 1,075)	(*n* = 206)	(*n* = 675)	(*n* = 553)	
Age (years)^a^	79.3 ± 9.3	79.1 ± 10.2	81.0 ± 9.0	79.4 ± 8.6	78.9 ± 8.6	0.036
Gender (%)						0.007
Males	1,390 (55.4%)	575 (53.5%)	113 (54.9%)	360 (53.3%)	342 (61.8%)	
Females	1,119 (44.6%)	500 (46.5%)	93 (45.1%)	315 (46.7%)	211 (38.2%)	
Residence (%)						<0.001
Rural	1,213 (48.3%)	690 (64.2%)	79 (38.3%)	283 (41.9%)	161 (29.1%)	
Town	1,296 (51.7%)	385 (35.8%)	127 (61.7%)	392 (58.1%)	392 (70.9%)	
Education (%)						<0.001
Illiterate	1,032 (41.1%)	523 (48.7%)	76 (36.9%)	298 (44.1%)	135 (24.4%)	
Literacy	1,477 (58.9%)	552 (51.3%)	130 (63.1%)	377 (55.9%)	418 (75.6%)	
Marital (%)						0.191
Married	1,434 (57.2%)	620 (57.7%)	110 (53.4%)	371 (55.0%)	333 (60.2%)	
Other	1,075 (42.8%)	455 (42.3%)	96 (46.6%)	304 (45.0%)	220 (39.8%)	
Health insurance (%)						<0.001
Yes	2,241 (89.3%)	983 (91.4%)	176 (85.4%)	614 (91.0%)	468 (84.6%)	
No	268 (10.7%)	92 (8.6%)	30 (14.6%)	61 (9.0%)	85 (15.4%)	
Living with family (%)						0.251
Cohabitation	2060 (82.1%)	871 (81.0%)	179 (86.9%)	556 (82.4%)	454 (82.1%)	
Living alone	449 (17.9%)	204 (19.0%)	27 (13.1%)	119 (17.6%)	99 (17.9%)	
Smoke (%)						0.084
Yes	623 (24.8%)	286 (26.6%)	59 (28.6%)	155 (23.0%)	123 (22.2%)	
No	1886 (75.2%)	789 (73.4%)	147 (71.4%)	520 (77.0%)	430 (77.8%)	
Drink (%)						0.151
Yes	588 (23.4%)	272 (25.3%)	40 (19.4%)	158 (23.4%)	118 (21.3%)	
No	1921 (76.6%)	803 (74.7%)	166 (80.6%)	517 (76.6%)	435 (78.7%)	
BMI (mean ± SD)	22.2 ± 3.9	21.9 ± 3.8	22.3 ± 4.3	22.3 ± 3.8	22.8 ± 4.0	<0.001
Diabetes (%)						<0.001
Yes	118 (4.7%)	32 (3.0%)	5 (2.4%)	36 (5.3%)	45 (8.1%)	
No	2,391 (95.3%)	1,043 (97.0%)	201 (97.6%)	639 (94.7%)	508 (91.9%)	
Heart disease (%)						<0.001
Yes	294 (11.7%)	92 (8.6%)	32 (15.5%)	80 (11.9%)	90 (16.3%)	
No	2,215 (88.3%)	983 (91.4%)	174 (84.5%)	595 (88.1%)	463 (83.7%)	
Cerebrovascular (%)						0.015
Yes	162 (6.5%)	53 (4.9%)	18 (8.7%)	43 (6.4%)	48 (8.7%)	
No	2,347 (93.5%)	1,022 (95.1%)	188 (91.3%)	632 (93.6%)	505 (91.3%)	
Self-reported healthy (%)						0.007
Good	1,517 (60.5%)	638 (59.3%)	126 (61.2%)	386 (57.2%)	367 (66.4%)	
Average	797 (31.8%)	354 (32.9%)	58 (28.2%)	227 (33.6%)	158 (28.6%)	
Poor	195 (7.8%)	83 (7.7%)	22 (10.7%)	62 (9.2%)	28 (5.1%)	
Depressive (%)						0.226
Yes	244 (9.7%)	98 (9.1%)	28 (13.6%)	68 (10.1%)	50 (9.0%)	
No	2,265 (90.3%)	977 (90.9%)	178 (86.4%)	607 (89.9%)	503 (91.0%)	
Sleep (%)						0.043
Good	1772 (70.6%)	754 (70.1%)	149 (72.3%)	456 (67.6%)	413 (74.7%)	
Average	503 (20.0%)	232 (21.6%)	40 (19.4%)	143 (21.2%)	88 (15.9%)	
Poor	234 (9.3%)	89 (8.3%)	17 (8.3%)	76 (11.3%)	52 (9.4%)	
Incident low psychological resilience	At/Above median: 1666 (66.4%) Below median: 843 (33.6%)	At/Above median: 621 (57.8%) Below median: 454 (42.2%)	At/Above median: 143 (69.4%) Below median: 63 (30.6%)	At/Above median: 473 (70.1%) Below median: 202 (29.9%)	At/Above median: 429 (77.6%) Below median: 124 (22.4%)	<0.001

a Continuous variables are expressed as means ± standard deviations and categorical variables are expressed as percentages. BMI, Body Mass Index.

### 3.2 Association between PA and PR

Table 3 presents the cross-sectional associations between physical activity (PA) patterns reported at baseline and low psychological resilience (PR). In the unadjusted model, the inactive-to-active and remain-active groups had lower odds of low PR than the remain-inactive group. The corresponding ORs were 0.52 (95% CI: 0.45–0.59) and 0.42 (95% CI: 0.36–0.49), respectively (both *p* < 0.001). These associations remained significant after adjustment for sociodemographic characteristics, lifestyle factors, and health-related variables. The adjusted ORs were 0.59 (95% CI: 0.51–0.69) for the inactive-to-active group and 0.61 (95% CI: 0.51–0.72) for the remain-active group (both *p* < 0.001). However, the association for the active-to-inactive group was not statistically significant in the fully adjusted model (OR = 0.94, 95% CI: 0.77–1.13; *p* = 0.486).

**Table 3:** Cross-sectional association between baseline PA and PR.^a^

PR	Model 1		Model 2		Model 3	
PA	OR (95%CI)	*p*	OR (95%CI)	*p*	OR (95%CI)	*p*
Remain inactive	Ref	–	Ref	–	Ref	–
Active to inactive	0.96 (0.81, 1.14)	0.647	1.05 (0.88, 1.25)	0.604	0.94 (0.77, 1.13)	0.486
Inactive to active	0.52 (0.45, 0.59)	<0.001	0.57 (0.50, 0.66)	<0.001	0.59 (0.51, 0.69)	<0.001
Remain active	0.42 (0.36, 0.49)	<0.001	0.54 (0.46, 0.64)	<0.001	0.61 (0.51, 0.72)	<0.001

a Logistic Regression.

Odds ratio; CI, confidence interval; PR, PR; PA, PA.

Model 1 = adjust for none; Model 2 = age + gender + residence + education + Health insurance +Living with family; Model 3 = Model 2 + smoking + drinking + Diabetes + heart + Cerebrovascular + Self reported health +Depressive + sleep.

The longitudinal analysis included 2,509 participants without low PR at baseline, of whom 843 (33.6%) had low PR at the 2014 follow-up. Table 4 presents the associations between PA patterns reported at baseline and incident low PR. After adjustment for sociodemographic characteristics, lifestyle factors, and health-related variables, all three PA groups had hazard ratios below 1 relative to the remain-inactive group. The adjusted HR was 0.72 (95% CI: 0.61–0.85; *p* < 0.001) for the inactive-to-active group and 0.59 (95% CI: 0.48–0.73; *p* < 0.001) for the remain-active group. The active-to-inactive group also showed an inverse association (HR = 0.73, 95% CI: 0.56–0.96; *p* = 0.023), which was not statistically significant in the cross-sectional analysis.

**Table 4:** Association between PA and PR during follow-up.^a^

PR	Model 1		Model 2		Model 3	
PA	HR (95%CI)	*p*	HR (95%CI)	*p*	HR (95%CI)	*p*
Remain inactive	Ref	–	Ref	–	Ref	–
Active to inactive	0.72 (0.56, 0.94)	0.016	0.73 (0.56, 0.95)	0.021	0.73 (0.56, 0.96)	0.023
Inactive to active	0.71 (0.60, 0.84)	<0.001	0.72 (0.61, 0.85)	<0.001	0.72 (0.61, 0.85)	<0.001
Remain active	0.53 (0.44, 0.65)	<0.001	0.56 (0.46, 0.69)	<0.001	0.59 (0.48, 0.73)	<0.001

a Cox Proportional Hazards Regression.

HR, Hazard Ratio; CI, confidence interval; PR, PR; PA, PA.

Model 1 = adjust for none; Model 2 = age + gender + residence + education + Health insurance + Living with family; Model 3 = Model 2 + smoking + drinking + Diabetes + heart + Cerebrovascular + Self-reported health + Depressive + sleep.

### 3.3 Subgroup analysis

To examine whether the associations between PA patterns and incident low PR differed across participant characteristics, we conducted exploratory subgroup analyses (Figure 2 and Supplementary Table S1). HR point estimates were below 1 across all examined strata, although several confidence intervals included 1. Compared with the remain-inactive group, the remain-active group showed an inverse association among participants aged <90 years (HR = 0.59, 95% CI: 0.47–0.74). This association was also observed among those aged ≥90 years (HR = 0.56, 95% CI: 0.33–0.93). The corresponding HRs were 0.56 (95% CI: 0.42–0.75) in males and 0.63 (95% CI: 0.47–0.85) in females. Inverse associations were also observed among smokers (HR = 0.55, 95% CI: 0.35–0.87) and alcohol drinkers (HR = 0.59, 95% CI: 0.36–0.95). No statistically significant PA-by-subgroup interactions were detected.

**Figure 2:**
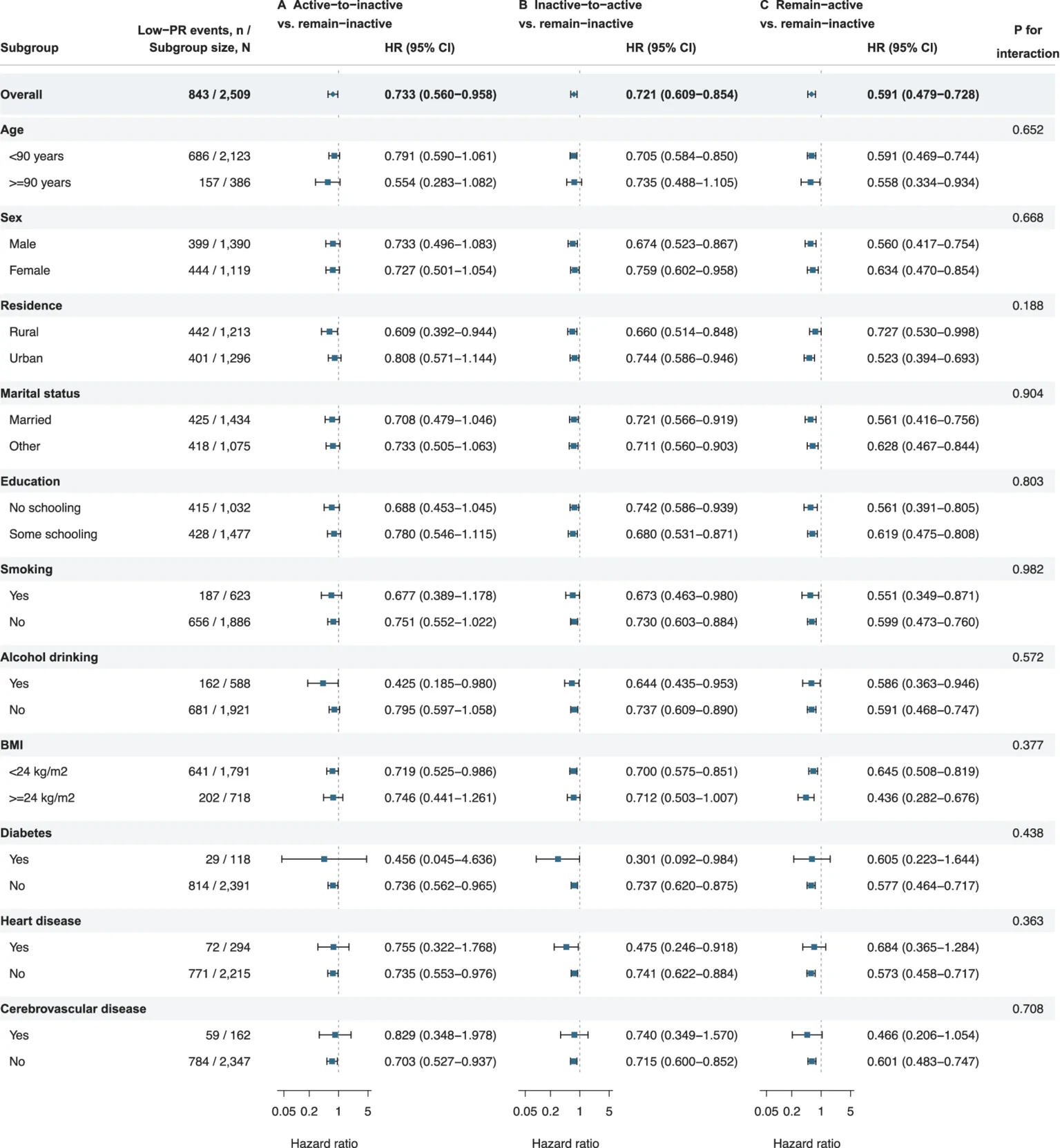
Subgroup analyses of the associations between baseline PA patterns and incident low PR. Subgroup models were adjusted for the covariates included in Model 3, except for the corresponding stratification variable. Age and BMI were omitted from the age- and BMI-stratified models, respectively.

### 3.4 Sensitivity analysis

To evaluate the robustness of our findings, we conducted two sensitivity analyses (Table 5). First, participants with missing covariate data were included using multiple imputation. Compared with the remain-inactive group, the active-to-inactive group had a lower hazard of incident low PR in Model 3 (HR = 0.78, 95% CI: 0.61–0.99; *p* = 0.039). The corresponding HRs were 0.72 (95% CI: 0.62–0.84; *p* < 0.001) for the inactive-to-active group and 0.62 (95% CI: 0.51–0.75; *p* < 0.001) for the remain-active group.

**Table 5:** Sensitivity analyses of the association between PA and PR during follow-up.^a^

PA	Model 1		Model 2		Model 3	
	HR (95% CI)	*p*	HR (95% CI)	*p*	HR (95% CI)	*p*
Included participants with missing data on covariates						
Remain inactive	Ref		Ref		Ref	
Active to inactive	0.77 (0.61, 0.97)	0.024	0.77 (0.61, 0.97)	0.029	0.78 (0.61, 0.99)	0.039
Inactive to active	0.70 (0.60, 0.81)	<0.001	0.71 (0.61, 0.83)	<0.001	0.72 (0.62, 0.84)	<0.001
Remain active	0.54 (0.45, 0.65)	<0.001	0.58 (0.48, 0.70)	<0.001	0.62 (0.51, 0.75)	<0.001
Excluding participants with baseline depressive symptoms						
Remain inactive	Ref		Ref		Ref	
Active to inactive	0.71 (0.54, 0.95)	0.021	0.70 (0.53, 0.94)	0.018	0.72 (0.54, 0.96)	0.025
Inactive to active	0.73 (0.61, 0.86)	<0.001	0.72 (0.60, 0.86)	<0.001	0.73 (0.61, 0.87)	<0.001
Remain active	0.54 (0.44, 0.67)	<0.001	0.56 (0.45, 0.70)	<0.001	0.59 (0.47, 0.74)	<0.001

a Cox Proportional Hazards Regression.

HR, Hazard Ratio; CI, confidence interval; PR, PR; PA, PA.

Model 1 = adjust for none; Model 2 = age + gender + residence + education + Health insurance + Living with family; Model 3 = Model 2 + smoking + drinking + Diabetes + heart + Cerebrovascular + Self-reported health + Depressive + sleep.

Second, excluding participants with baseline depressive symptoms yielded results broadly consistent with the main analysis. Compared with the remain-inactive group, all three PA groups had lower hazards of incident low PR in Model 3. The HRs were 0.72, 0.73, and 0.59 for the active-to-inactive, inactive-to-active, and remain-active groups, respectively, with corresponding 95% CIs of 0.54–0.96, 0.61–0.87, and 0.47–0.74. All three associations remained statistically significant, with *p* = 0.025 for the active-to-inactive group and *p* < 0.001 for the other two groups.

## 4 Discussion

Using data from the CLHLS, we examined the cross-sectional and longitudinal associations between PA patterns reported at baseline and low PR. Compared with the remain-inactive group, the inactive-to-active and remain-active groups had lower odds of prevalent low PR, with adjusted ORs of 0.59 and 0.61, respectively. These groups also had lower hazards of incident low PR during follow-up, with adjusted HRs of 0.72 and 0.59, respectively. The active-to-inactive group showed an inverse association in the longitudinal analysis but not in the cross-sectional analysis. No statistically significant interactions were detected in the exploratory subgroup analyses. Estimates from both sensitivity analyses were broadly consistent with the main findings.

Our results align with the broad consensus that PA benefits mental health. Across both cross-sectional and longitudinal analyses, we observed a positive association between PA and PR, with physically active older adults demonstrating greater resilience than their inactive counterparts. Some studies (Resnick et al., 2019; Wermelinger Ávila et al., 2022) suggest an indirect relationship, with PA acting as a moderator, whereas others (Perna et al., 2012; Veldema and Jansen, 2019) report a direct positive association between higher PA levels and stronger PR. As noted by Toth (Toth et al., 2024) in their systematic review, cross-sectional designs dominate the existing literature on PA and PR, while prospective cohort evidence remains scarce. This predominance of cross-sectional studies has limited conclusions regarding temporality and causality. Our study adds longitudinal observational evidence on the association between PA and PR; however, causal relationships cannot be established.

Previous studies have demonstrated that physical activity plays a crucial role in maintaining and enhancing psychological resilience in older adults, with this association observed across diverse cultural contexts. Majnarić et al. (2021) reported that lower psychological resilience in older adults is associated with accelerated aging and a higher incidence of health complications, highlighting the key role of health-promoting behaviors, such as physical activity, in mitigating these adverse effects and directly reducing disease risk. Similarly, Zach et al. (2021) found that while physical activity is associated with higher resilience levels, older adults generally exhibit lower resilience compared with younger populations, potentially due to age-related declines in physical activity. Regarding activity patterns, Martínez-Moreno et al. (2020) reported that only 43.6% of older adults engaged in physical activity more than 3 days per week. Consistent with our findings, they also confirmed that older adults who participate in regular physical activity exhibit significantly higher resilience scores. Furthermore, Kukihara et al. (2018) investigated older adults in Japan and found that physically active individuals not only demonstrated higher psychological resilience compared with their less active peers but also exhibited greater life morale and lower risk of depressive symptoms.

The beneficial effect of physical activity on psychological resilience is supported by multilevel mechanisms spanning neurobiological, individual, and psychosocial domains. At the neurobiological level, regular physical activity provides a foundation for resilience. It is one of the most effective modulators of the hypothalamic–pituitary–adrenal (HPA) axis, optimizing cortisol responses to stress and reducing long-term physiological burden (Rimmele et al., 2007; Gerber and Pühse, 2009). Exercise also upregulates neurotrophic factors, such as brain-derived neurotrophic factor, which enhance neural plasticity in the hippocampus and prefrontal cortex, thereby fundamentally improving emotion regulation and cognitive flexibility (Cotman et al., 2007; Duman and Aghajanian, 2012)—core neurobiological substrates of high psychological resilience. This active neuroplastic process provides both the biological potential and stability necessary for the development of resilience. At the individual level, regular physical activity may strengthen self-efficacy through repeated successful experiences, which may contribute to psychological resilience (Oliveira et al., 2025). At the social level, supportive environments, access to exercise opportunities, health-related information, and social support may facilitate sustained participation in physical activity and contribute to psychological well-being (Sherwood and Jeffery, 2000; Kaczynski and Henderson, 2007; Cutler and Lleras-Muney, 2010; Berkman et al., 2011) However, no statistically significant PA-by-subgroup interactions were detected in the present study. Therefore, the subgroup-specific estimates should be interpreted cautiously and should not be considered evidence that the association between PA and PR differs across demographic or health-related subgroups.

These findings have several implications for public health practice and healthy-aging policy. The observed associations suggest that physical activity may be relevant to broader strategies aimed at supporting psychological well-being and resilience in later life. In particular, the consistent associations observed for both becoming active and remaining active may inform community-based health promotion programs that seek to reduce physical inactivity among older adults and support sustained participation in physical activity. The findings may also provide useful evidence for integrating physical activity promotion into broader healthy-aging and mental-health promotion initiatives for older adults.

This study is the first to explore the longitudinal relationship between dynamic PA changes and PR, providing insights for active and healthy aging. However, several limitations should be acknowledged. First, PA was assessed using a single, simple self-reported question, which did not capture intensity, duration, or type, potentially introducing measurement error and precluding dose–response analyses. Second, because this study was a secondary analysis of observational cohort data, causal relationships between PA and PR cannot be established. Although the longitudinal analysis provides temporal information, residual confounding (e.g., genetic predisposition, personality traits) and reverse causality (i.e., individuals with higher PR being more likely to maintain activity) cannot be entirely excluded. Third, the study sample comprised community-dwelling older adults in China, and cultural context and common forms of PA may limit generalizability. Finally, some subgroup categories had small sample sizes, which may have affected statistical power. These limitations point to several priorities for future research. Future studies should use more detailed and objective measures of PA, including intensity, duration, frequency, and type, to examine potential dose–response relationships. Intervention studies are also needed to determine whether changes in PA can causally improve PR. In addition, larger and more diverse cohorts would help clarify whether these associations differ across population subgroups and cultural contexts.

## 5 Conclusion

PA patterns reported at baseline were associated with prevalent and incident low PR among Chinese older adults. Compared with the remain-inactive group, the inactive-to-active and remain-active groups had lower odds of prevalent low PR and lower hazards of incident low PR, while the active-to-inactive group showed a lower hazard only in the longitudinal analysis. These observational findings identify PA as a potentially modifiable correlate of psychological resilience, although intervention studies are needed to assess causality.

## Data Availability

Publicly available datasets were analyzed in this study. This data can be found here: https://opendata.pku.edu.cn.
