# Supplementary material for: Association between physical activity and psychological resilience in older adults: an analysis of a prospective cohort study in China
Source: Front Psychol. 2026 Oct 9;17:1958017. doi: 10.3389/fpsyg.2026.1958017 (PMC13690944; doi:10.3389/fpsyg.2026.1958017)
Supplement: Supplementary file 1 [file Table_1.DOCX]

Supplementary Table S1. Subgroup analyses of the associations between baseline PA patterns and incident low psychological resilience

| **Subgroup** | **Category** | **Events, n / Total, N** | **Active-to-inactive HR (95% CI)** | **Inactive-to-active HR (95% CI)** | **Remain-active HR (95% CI)** | **P for interaction** |
| --- | --- | --- | --- | --- | --- | --- |
| Overall | — | 843 / 2,509 | 0.733 (0.560–0.958) | 0.721 (0.609–0.854) | 0.591 (0.479–0.728) | — |
| Age | <90 years | 686 / 2,123 | 0.791 (0.590–1.061) | 0.705 (0.584–0.850) | 0.591 (0.469–0.744) | 0.652 |
|  | ≥90 years | 157 / 386 | 0.554 (0.283–1.082) | 0.735 (0.488–1.105) | 0.558 (0.334–0.934) |  |
| Sex | Male | 399 / 1,390 | 0.733 (0.496–1.083) | 0.674 (0.523–0.867) | 0.560 (0.417–0.754) | 0.668 |
|  | Female | 444 / 1,119 | 0.727 (0.501–1.054) | 0.759 (0.602–0.958) | 0.634 (0.470–0.854) |  |
| Residence | Rural | 442 / 1,213 | 0.609 (0.392–0.944) | 0.660 (0.514–0.848) | 0.727 (0.530–0.998) | 0.188 |
|  | Urban | 401 / 1,296 | 0.808 (0.571–1.144) | 0.744 (0.586–0.946) | 0.523 (0.394–0.693) |  |
| Marital status | Married | 425 / 1,434 | 0.708 (0.479–1.046) | 0.721 (0.566–0.919) | 0.561 (0.416–0.756) | 0.904 |
|  | Other | 418 / 1,075 | 0.733 (0.505–1.063) | 0.711 (0.560–0.903) | 0.628 (0.467–0.844) |  |
| Education | No schooling | 415 / 1,032 | 0.688 (0.453–1.045) | 0.742 (0.586–0.939) | 0.561 (0.391–0.805) | 0.803 |
|  | Some schooling | 428 / 1,477 | 0.780 (0.546–1.115) | 0.680 (0.531–0.871) | 0.619 (0.475–0.808) |  |
| Smoking | Yes | 187 / 623 | 0.677 (0.389–1.178) | 0.673 (0.463–0.980) | 0.551 (0.349–0.871) | 0.982 |
|  | No | 656 / 1,886 | 0.751 (0.552–1.022) | 0.730 (0.603–0.884) | 0.599 (0.473–0.760) |  |
| Alcohol drinking | Yes | 162 / 588 | 0.425 (0.185–0.980) | 0.644 (0.435–0.953) | 0.586 (0.363–0.946) | 0.572 |
|  | No | 681 / 1,921 | 0.795 (0.597–1.058) | 0.737 (0.609–0.890) | 0.591 (0.468–0.747) |  |
| BMI | <24 kg/m² | 641 / 1,791 | 0.719 (0.525–0.986) | 0.700 (0.575–0.851) | 0.645 (0.508–0.819) | 0.377 |
|  | ≥24 kg/m² | 202 / 718 | 0.746 (0.441–1.261) | 0.712 (0.503–1.007) | 0.436 (0.282–0.676) |  |
| Diabetes | Yes | 29 / 118 | 0.456 (0.045–4.636) | 0.301 (0.092–0.984) | 0.605 (0.223–1.644) | 0.438 |
|  | No | 814 / 2,391 | 0.736 (0.562–0.965) | 0.737 (0.620–0.875) | 0.577 (0.464–0.717) |  |
| Heart disease | Yes | 72 / 294 | 0.755 (0.322–1.768) | 0.475 (0.246–0.918) | 0.684 (0.365–1.284) | 0.363 |
|  | No | 771 / 2,215 | 0.735 (0.553–0.976) | 0.741 (0.622–0.884) | 0.573 (0.458–0.717) |  |
| Cerebrovascular disease | Yes | 59 / 162 | 0.829 (0.348–1.978) | 0.740 (0.349–1.570) | 0.466 (0.206–1.054) | 0.708 |
|  | No | 784 / 2,347 | 0.703 (0.527–0.937) | 0.715 (0.600–0.852) | 0.601 (0.483–0.747) |  |

Note: The remain-inactive group served as the reference category. Events represent the number of incident low-PR cases, and N represents the total number of participants in each subgroup. HR, hazard ratio; CI, confidence interval; PA, physical activity; PR, psychological resilience; BMI, body mass index. Subgroup models were adjusted for the covariates included in Model 3, except for the corresponding stratification variable.
